# Polymer Particles Bearing Recombinant LEL CD81 as Trapping Systems for Hepatitis C Virus

**DOI:** 10.3390/pharmaceutics13050672

**Published:** 2021-05-07

**Authors:** Dmitry Polyakov, Ekaterina Sinitsyna, Natalia Grudinina, Mariia Antipchik, Rodion Sakhabeev, Viktor Korzhikov-Vlakh, Mikhail Shavlovsky, Evgenia Korzhikova-Vlakh, Tatiana Tennikova

**Affiliations:** 1 Institute of Experimental Medicine, Acad. Pavlov Street 12, 197376 St. Petersburg, Russia; ravendoctor@mail.ru (D.P.); strangecatnap@gmail.com (N.G.); helm505@mail.ru (R.S.); mmsch@rambler.ru (M.S.); 2 Institute of Macromolecular Compounds, Russian Academy of Sciences, Bolshoy pr. 31, 199004 St. Petersburg, Russia; kat_sinitsyna@mail.ru (E.S.); volokitinamariya@yandex.ru (M.A.); 3 Institute of Chemistry, Saint-Petersburg State University, Universitetsky pr. 26, 198504 St. Petersburg, Russia; v.korzhikov-vlakh@spbu.ru

**Keywords:** recombinant CD81-streptavidin fusion protein, trapping system for HCV, poly(lactic acid)-based micro- and nanoparticles, interaction of LEL CD81 with E2 protein, virus-mimicking particles

## Abstract

Hepatitis C is one of the most common social diseases in the world. The improvements in both the early diagnostics of the hepatitis C and the treatment of acute viremia caused by hepatitis C virus are undoubtedly an urgent task. In present work, we offered the micro- and nanotraps for the capturing of HCV. As a capturing moiety, we designed and synthesized in *E. coli* a fusion protein consisting of large extracellular loop of CD81 receptor and streptavidin as spacing part. The obtained protein has been immobilized on the surface of PLA-based micro- and nanoparticles. The developed trapping systems were characterized in terms of their physico-chemical properties. In order to illustrate the ability of developed micro- and nanotraps to bind HCV, E2 core protein of HCV was synthesized as a fusion protein with GFP. Interaction of E2 protein and hepatitis C virus-mimicking particles with the developed trapping systems were testified by several methods.

## 1. Introduction

Hepatitis C is one of the major social diseases. Today, about 2% of the world’s population suffer from the infection by hepatitis C virus (HCV) [1]. The current treatment is based on administration of PEGylated alpha-interferon (PEG-IFN) and ribavirin (RBV) with directly acting viral protease inhibitors [2]. The therapy is limited by low efficiency and many side-effects. One of the ways to reduce the morbidity is the vaccination of the population. Currently, the efforts of many research groups are focused on the development of effective HCV vaccines [1,3] that are still absent on the world market. To overcome the drawbacks of PEG-IFN/RBV treatment, the development of a new, specifically targeted antiviral therapy for HCV can be a matter of choice [4]. In particular, the achievements of nanotechnology as targeted delivery systems for HCV treatment are summarized in a recent review [5]. Besides the development of vaccines and targeted delivery systems, an actual task is a necessity to reduce the toxic effects under acute conditions like viremia as well as to increase the sensitivity level of existing methods for early diagnostics.

In current research, we suggest an approach for blocking of HCV using specially developed micro- or nanotraps. Trapping systems represent a novel field in the development of nanomedicines and bioanalytical approaches, and they are designed to selectively trap the target virus, infectious virions, and viral proteins [6]. Micro- and nanotraps can be applied for two main goals: (1) at acute viremia, to trap the viruses and virus pathogens in blood and, finally, to eliminate them through phagocytosis; (2) at early stage of disease, to concentrate virus particles for further assay methodologies. While the application of micro- and nanotraps in vivo is poorly studied, the utilization of trapping systems to enhance the detection of virus disease at early stages is a widespread technique [7]. In particular, the use of trapping systems in the diagnosis of HIV-1 [8], Rift Valley fever virus (RVFV) [9], Venezuelan equine encephalitis virus (VEEV) [10], human coronavirus and influenza virus from different complex matrices like saliva, nasal fluid, and nasal aspirates [11], retrovirus [12], as well as Zika, chikungunya and dengue viruses in urine [13], have recently been reported. In addition, the nanotraps for highly efficient purification of intact foot-and-mouth disease virus (FMDV) to manufacture inactivated vaccine have been developed [14]. The proteins capturing by nanotraps for isolation [15] or blocking their undesirable biological function [16] is also a known approach.

**Figure 1:**
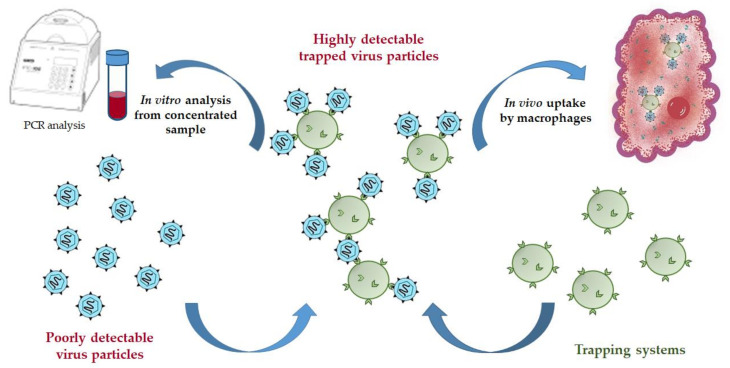
Scheme of possible application of trapping systems for virus capturing.

In this work, we have designed the micro- and nanotraps capable of HCV E2-protein binding. Such trapping systems can serve as practically useful tools for both in vitro concentration of HCV from human blood for further reliable detection or in vivo application to reduce acute HCV-caused viremia (Figure 1). The detection of HCV on the early stages is problematic due to very low concentration of the virus in human blood. The proposed trapping systems based on microparticles could capture virions being added into the sample volume. In turn, the virions bound to the functionalized polymer particles are easily concentrated by centrifugation, and thus the sample containing many times a greater number of virions can be used for further detection by a set of existed analytical methods. In the second case, the trapping systems could be applied in vivo to treat acute viremia to capture HCV and to reduce their concentration in blood due to further phagocytosis of the formed enlarged complexes.

In order to prepare the trapping systems for HCV, we have applied polymer micro- and nanoparticles based on biodegradable poly(lactic acid) (PLA) modified with fragment of human receptor specific to HCV. The application of antibodies obtained against hepatitis C to prepare trapping systems seems to be not very effective because of the high genetic variability of HCV genome that gives rise to escape mutants. However, all genotypes of HCV save the ability to bind human CD81 receptor [17]. The CD81 protein consists of 236 amino acids (MW 25.8 kDa). The N-and C-terminal sites are localized in the cytoplasm. It is known that HCV penetrates into the hepatocytes via receptor-mediated endocytosis [18,19]. At first step, HCV envelope glycoprotein E2 recognizes the ‘‘head’’ subdomain of the large extracellular loop (LEL) of CD81 membrane receptor [20,21,22] and then later slowly penetrates the cell. Thus, the CD81 seems to be the best candidate to prepare stable trapping system for HCV.

Following the idea to obtain the HCV trapping systems by the modification of PLA-based particles with CD81, we designed and synthesized a recombinant fusion protein of LEL CD81 with streptavidin (SA) (LEL CD81-SA). In this case, streptavidin serves as a spacer between the surface of the microparticles and the target protein. Moreover, it can be used for streptavidin-based HCV assays. PLA-based micro- and nanoparticles were used as a base for immobilization of LEL CD81-SA to prepare the final HCV trapping systems. The efficiency of trapping system bearing LEL CD81 to bind E2 of HCV must be confirmed. Since the work with HCV is dangerous and requires special conditions, the applicability of the developed systems to trap HCV was studied with the use of specially constructed hepatitis C virus-mimicking nanoparticles (HC VMP). HC VMP were prepared using PLA-based nanoparticles of 90 nm (close to HCV size [23]) and recombinant E2 protein.

## 2. Materials and Methods

### 2.1. Materials

d,l-lactide, poly(ethylene glycol) methyl ester (*M_n_* = 5000), stannous octoate, *N*-(3-dimethylaminopropyl)-*N*′-ethylcarbodiimide hydrochloride (EDC), polyoxyethylene-polyoxypropylene triblock copolymer (Lutrol F68), poly(vinyl alcohol) (*M_w_ =* 130,000), 2-(N-morpholino)ethanesulfonic acid (MES), and N-hydroxysuccinimide (NHS) were acquired from Sigma-Aldrich (Munich, Germany). Sodium dodecylsulfate (SDS) was the product of Carl Roth (Karlsruhe, Germany). Acetonitrile was purchased from J.T. Baker (Phillipsburg, NJ, USA). Hexane, toluene, methanol and tetrahydrofuran (THF) were obtained from Vecton Ltd. (St. Petersburg, Russia). All solvents were distilled prior to use. For preparation of buffers, the salts were purchased in Sigma-Aldrich (Munich, Germany). Before application, all buffer solutions were filtered using membranes with pore size of 0.45 µm (Millipore-Merck, Darmstadt, Germany). The polystyrene standards used for column calibration in size-exclusion chromatography (SEC) were acquired from Waters (Milford, MS, USA). The following reagents were used for production, purification, and analysis of the proteins: Nuclease-free water (Ambion, Austin, TX, USA), ethidium bromide (Serva, Heidelberg, Germany), isopropyl-β-d-1-thiogalactopyranoside (IPTG) (Fisher BioReagents, Waltham, MS, USA), imidazole (Chemical Line, St. Petersburg, Russia), 2-mercaptoethanol (BASF, Ludwigshafen, Germany), Ni-chelate sorbent (Invitrogen, Waltham, MS, USA), selective agarose medium (Conda, Madrid, Spain), and ampicillin (Sintez, Kurgan, Russia).

### 2.2. Synthesis of LEL CD81-SA Fusion Protein

#### 2.2.1. Production of LEL CD81-SA in *E. coli*

The design of genetic constructs is presented in Figure S1 of Supplementary Materials. The strain of *E. coli* DH5(α) was used to develop the initial vector of pET SA 2-7. To insert the nucleotide sequences, the “sticky ends” obtained by processing the vector with the NdeI restriction endonuclease were used. The source vector was then used to obtain a genetic construct encoding streptavidin with LEL CD81. The resulting pET SA 2-7-LEL CD81 construct was transformed into *E. coli* BL21(DE3) cells. From 100 to 300 ng of pET SA 2-7-LEL CD81 plasmid DNA was added to 100 µL of *E. coli* BL21(DE3) cells and incubated in ice for 15 min. To perform the heat shock, the cell suspension was incubated for 5 min at 42 °C in a water bath, followed by incubation in ice for 2 min. After that, 900 µL of LB medium was added to the test tube and incubated for 1 h at 37 °C. The cells were seeded on cups containing agarized culture medium with ampicillin (100 µg/mL). The transformed cells are resistant to these antibiotics and retain their viability, while the original cells die. The resulting transformants were called as *E. coli* BL21 (DE3)/pET SA 2-7-LEL CD81. They were sown in 10 mL of culture medium with the addition of ampicillin (100 µg/mL) and left to grow overnight at 37 °C. After 18 h, the inoculate was poured into 500 mL of culture medium. For the selection of plasmid-transformed cells, the antibiotic ampicillin was added (100 µg/mL). The cells were grown at 37 °C under aeration conditions to a density of A_600_ = 0.6–1.0. Then, protein synthesis was induced by adding IPTG (60 mg per 500 mL of medium), and cultivation was continued overnight at 37 °C. The resulting cell biomass was then used to isolate the protein from various cell fractions.

#### 2.2.2. Separation and Purification of LEL CD81-SA

Bacterial cells were separated from the culture medium by centrifugation for 30 min at 3500 rpm. The deposited cells were resuspended in PBS and centrifuged at a speed of 4000 rpm for 15 min, and then destroyed using an ultrasonic disintegrator under cooling conditions with the addition of glass beads (GlassBeads, 500 µm, Sigma-Aldrich, Moscow, Russia). After removing the glass beads, the supernatant and the cell mass were centrifuged at a speed of 13,500 rpm for 15 min. The supernatant filtered through a paper filter was subjected to chromatographic purification on a Ni-agarose column (column volume 1.5 mL), according to the standard protocol of the sorbent manufacturer (Invitrogen, Waltham, MS, USA). The column was loaded with 30 mL of supernatant in 10 mM imidazole, 0.3 M NaCl, 50 mM Tris-HCl, pH 8.0. Desorption was carried out by adding 5 mL of a solution containing 200 mM imidazole, 0.3 M NaCl, 50 mM Tris-HCl, pH 8.0. The mobile phase flow rate was 0.3 mL/min. The target protein was detected using polyacrylamide gel-electrophoresis (PAGE).

The precipitate of destroyed cells remaining after isolation from the soluble cell fraction was washed with PBS, dissolved in solution of 8 M urea or 6 M guanidine chloride (pH 1.5) and left for 1 h. The precipitate was then centrifuged at 12,000 rpm for 15 min and purified as it was described above.

Fractions resulting from protein purification on the Ni-agarose were analyzed by gel-electrophoresis in 5–12% polyacrylamide gel in a presence of sodium dodecyl sulfate (SDS PAGE). The Coomassie staining was applied for protein visualization. The standard protocol can be found elsewhere [24,25].

### 2.3. Synthesis of Chimeric E2 core-GFP Protein in E. coli

A combined expression genetic construct including an E2 core gene and the superfolder GFP gene was created. The E2 core cDNA was prepared by PCR using following primers: 5′-ctggaattcCAGCTGATCAACACC-3′ and 5′-ctgagctcAGACTGGAAGTACAGGTTTTCGTTGCAAGCAGCTTC-3′. The design of the plasmid was made in such a way that the encoding portion of E2 was followed with a sequence encoding of 6-His necessary for affinity purification of the protein on Ni-agarose gel. As a result, a 738 b.p. product was obtained, which, after treatment with restriction endonucleases EcoRI and SacI was inserted into the plasmid pTRC99a-superfolder GFP [26,27], treated with the same restriction endonucleases. Sequencing showed that the finished plasmid included the E2 core gene and the superfolder gene GFP in one reading frame.

The target protein (E2 core, 412–645 a.a.) was synthesized in *E. coli* strain BL (DE3) cells and isolated on Ni-agarose column. Protein expression was induced with the addition of IPTG (0.25 mmol) to OD_600_ of 0.6. Induction was continued for 16 h at 37 °C. After that, the cells were centrifuged, washed with saline solution, destroyed by ultrasonication, and then centrifuged to separate the soluble and insoluble cell fractions. The insoluble cell fraction was dissolved in 8 M urea.

The E2 core-GFP (E2-GFP) from insoluble cell fractions was performed also using Ni-agarose column. For this, the column was loaded with 30 mL of inclusion bodies dissolved in 8 M urea, 10 mM imidazole, 0.3 M NaCl, 50 mM Tris-HCl, pH 8.0. Desorption was carried out by adding 5 mL of a solution containing 8 M urea, 200 mM imidazole, 0.3 M NaCl, 50 mM Tris-HCl, pH 8.0. The mobile phase flow rate was 0.3 mL/min. The target protein in buffer containing urea could be detected visually by green fluorescence. Protein renaturation was carried out by step-wise dialysis with a decrease in the urea concentration from 7 to 0 M (7, 6, 5, 4, 3, 2, 1 and finally 0 M) in 50 mM Tris-HCl buffer, pH 8.0, at constant stirring. Each stage was carried out for 4–16 h. Purified protein was analyzed 5–12% SDS gel-electrophoresis in polyacrylamide gel. The Coomassie staining was applied for protein visualization. The standard protocol can be found elsewhere [24,25].

### 2.4. Synthesis and Characterization of poly(d,l-lactic acid) and poly(ethylene glycol)-b-poly(d,l-lactic acid) (PEG-b-PLA)

d,l-lactide was purified by recrystallization from toluene and washing with hexane, then the monomer was dried in vacuum at 25 °C for 16 h. The synthesis of poly(d,l-lactic acid) (PLA) was carried out by stannous octoate catalyzed ring-opening polymerization in bulk as previously published [28]. Briefly, the dry Schlenk tube was charged with monomer and the solution of stannous octoate in hexane was added to get monomer/catalyst molar ratio equal to 200. Then, the solvent was removed under vacuum. The polymerization was performed using magnetic stirrer MR Hei-Mix S (Heidolph, Schwabach, Germany) at 135 °C for 1 h. The reaction was quenched by ice cooling. The crude polymer product was dissolved in chloroform and precipitated in the excess of cold methanol. For obtaining of pure polymer the precipitation was performed twice. Finally, the product dried in vacuum at 35 °C. The polymer yield varied from 87 to 93%.

The synthesis of PLA block copolymers with poly(ethylene glycol) methyl ester (PEG-5000, *M_n_* = 5000) was performed by similar way. In this case, PEG-5000 was used as initiator along with stannous octoate as catalyst. The lactide/catalyst molar ratio was equal to 200. The molar ratio of monomer/PEG was 1500. The reaction was carried out at 135 °C for 1.5 h.

Molecular weights and dispersity of polymers obtained were determined by size-exclusion chromatography (SEC). The analysis was carried out on Shimadzu HPLC system with refractometric detector RID-10A (Shimadzu, Tokyo, Japan) and using Waters Styragel HMW 6E 7.8 × 300 mm column (Waters, Milford, MS, USA) at 40 °C. Tetrahydrofuran (THF) was used as eluent, and mobile phase flow was 0.3 mL/min. The calibration curve was built using polystyrene standards that covered a molecular weight range from 2000 to 144,000. The calculations were caried out with GPC LC Solutions software (Shimadzu, Tokyo, Japan). The characteristics of synthesized PLA were *M_w_* = 24,000, *M_n_* = 17,000, *Đ* = 1.4. The characteristics of obtained PEG-*b*-PLA were *M_w_* = 34,000, *M_n_* = 29,000, *Đ* = 1.2.

### 2.5. Preparation of PLA Microparticles and PEG-b-PLA Nanoparticles

PLA-based microparticles (PLA MPs) were obtained by the single emulsion technique using the protocol developed previously [28]. Briefly, 300 mg of PLA were dissolved in 6.0 mL of dichloromethane and stirred during 16 h to obtain the 5% (*w/v*) solution. The organic phase was injected with velocity 120 mL/min using syringe infusion pump Instilar 1438 (Dixion, St. Petersburg, Russia) into 60 mL of water phase containing 1% of sodium dodecylsulfate (SDS) and 3% of Lutrol F68 and dispersed via simultaneous action of ultrasound homogenizer (Sonopuls HD2070, Bandelin, Berlin, Germany) for 3 min with power 15% and 30 s with power 33% and magnetic stirrer MR Hei-Mix S (Heidolph, Schwabach, Germany) at 750 rpm. Then, the obtained emulsion was diluted with 180 mL of 1% PVA on magnetic stirrer at 800 rpm. In order to form particles suspension, dichloromethane was removed with rotary evaporator (Hei-VAP Precision ML/G3B (Heidolph, Schwabach, Germany) during 2 h, the pressure was changed in the range from 400 to 100 mbar. The particles were centrifuged at 10,000× *g* for 10 min, washed three times with distilled water. The yield of particles was 85%. Polymer particles were stored in distilled water in the refrigerator at 4 °C until further use.

The PEG-*b*-PLA-based nanoparticles (PEG-*b*-PLA NPs) were obtained by the nanoprecipitation technique. In this case, a solution of the PEG-*b*-PLA in acetonitrile with a concentration of 0.5 mg/mL was slowly introduced into water used as a precipitator. The ratio of the organic phase to water was 1 to 5. The nanoparticles were formed under magnetic stirring at 900 rpm. To remove organic solvent from the system, the suspension was incubated at 25 °C for 16 h. The yield of polymer particles was 94%.

In both cases, the average hydrodynamic diameter and index of polydispersity of obtained micro- and nanoparticles were evaluated using the dynamic light-scattering instrument (DLS, Zetasizer NanoZS, Malvern, Malvern, UK).

### 2.6. Preparation of HC Micro- and Nanotraps and HC VMPs

PLA microparticles and PEG-*b*-PLA nanoparticles were modified with LEL CD81-SA or with its Cy3-labeled form to prepare micro- and nanotraps, respectively. In addition, to prepare a control system PLA-based microparticles were modified with model protein, namely soybean trypsin inhibitor labeled with Cy3 (SBTI-Cy3). PEG-*b*-PLA nanoparticles were modified with E2-GFP to prepare HC VMPs. The scheme of modification with proteins was similar for both PLA microparticles and PEG-*b*-PLA nanoparticles.

Initially, the carboxylic groups were generated at PLA microparticles’ surface via partial hydrolysis of polymer ester bonds with sodium hydroxide solution. For this, 150 mg of PLA microparticles were firstly treated with 1.5 mL of 0.1 M NaOH for 30 min at 22 °C. To finish the process of polyester hydrolysis, the particles were centrifuged at 10,000× *g* for 7 min, washed with distilled water and sonicated for 5 s with ultrasound homogenizer. The washing procedure was repeated 3 times. In the case of PEG-*b*-PLA nanoparticles, 0.01 M NaOH was applied for hydrolysis and the duration of treatment was 15 min at 22 °C. The suspension of nanoparticles was purified by dialysis against water for 1.5 h.

The formed carboxylic groups were activated with NHS and EDC. The reaction was run in 0.05 M MES buffer, pH 5.6, for 30 min. In the case of microparticles, the activated particles were transferred into the water via three times centrifugation/decantation/resuspension in water while the nanoparticles were purified by dialysis against water. The protein solution (CD81-SA-Cy3, SBTI-Cy3 or E2-GFP) in 0.0125 M Na-borate buffer, pH 8.4, was added to activated particles. The reaction suspension was incubated at 25 °C for 2 h for microparticles and 1.5 h for nanoparticles.

After that, non-bound protein was removed as described above, and the prepared microtraps were resuspended in 0.01 M PBS, pH 7.4. For nanoparticles, non-bound protein was removed with the use of ultrafiltration trough the membranes with MWCO 100,000. The amount of unbound E2-GFP or LEL CD81-SA-Cy3/SBTI-Cy3 was measured spectrophotometrically at 490/550 nm.

The amount of bound protein was calculated as difference between initial amount of protein, amount of protein determined in supernatant after reaction and amount of protein in washing solutions:

*Q_imm_* = *Q_init_.* − *Q_supern_.* − *Q_wash_,* (1)

where *Q_imm_* is an amount of protein immobilized into the particles (mg), *Q_init_* is an initial amount of protein taken for immobilization (mg), *Q_supern_* is an amount of protein in supernatant after the reaction (mg), *Q_wash_* is an amount of protein determined in washing solutions (mg). The quantity of labeled protein was found according to calibration curve built for corresponding proteins.

The micro- and nanotraps, as well as HC VMPs in 0.01 PBS, pH 7.4, were stored at 4 °C.

### 2.7. Interaction of E2–GFP with Trapping Systems

#### 2.7.1. Interaction of E2-GFP with Microtraps at Static Conditions

E2-GFP was added to 0.5 mL of suspension of microtraps with concentration of microparticles equal to 0.1 mg/mL in 50 mM Tris-HCl, pH 8.0. The final concentration of E2-GFP was 0.01–0.1 mg/mL. In the control experiment, instead of E2-GFP, the same molar amount of GFP was used. The resulting mixtures were incubated for 1 h at 22 °C at constant stirring. After that, the PLA microparticles were precipitated by centrifugation at 3000 rpm for 15 min, washed two times in 50 mM Tris-HCl, pH 8.0, and carefully resuspended in 0.5 mL of the same buffer. The resulting suspension was analyzed by SDS PAGE and Laser Scanning Confocal Microscopy. The images were captured using an inverted confocal laser scanning microscope LSM 510 Meta (Zeiss, Jena, Germany), with lens EC Plan-Neofluar 10×/0.3 (Zeiss, Jena, Germany). Laser with a wavelength of 488 nm was used to excite the fluorescence, a 505–550 nm filter was used to detect GFP fluorescence. In transmitted light images were obtained using the Differential Interference Contrast (DIC) method.

#### 2.7.2. Interaction of E2-GFP with Microtraps at Dynamic Conditions

250 mg of CNBr-activated Sepharose 4B (Sigma-Aldrich, Munich, Germany) was washed in 50 mL of 1–2% HCl, pH 3.0, for 40 min. After that the resin was washed with cold water. LEL CD81-protein G (1.6 mg) was incubated with CNBr-activated Sepharose overnight at 5 °C in 0.1 M Na-carbonate buffer (pH 8.3), containing 0.5 mol/L NaCl. The obtained Sepharose gel (1 mL) with immobilized LEL CD81-SA was packed into a column and washed with PBS (20 mL) to eliminate the unbound protein. The mobile phase flow rate was 0.3 mL/min. Then, the protein solution containing the E2-GFP in PBS (pH 7.4) was loaded onto the column. To remove the non-specific binding, the column was washed with the excess of PBS containing 1 mol/L NaCl until the absence of the GFP fluorescence in washing solutions. The sample of Sepharose was taken and analyzed by confocal microscopy as described above. The final elution of the E2-GFP from affinity complex was carried out with 0.3 M Tris-glycine buffer, pH 9.5.

### 2.8. Interaction of HC VMPs with Nanotraps

The suspensions of HC VMPs and nanotraps in 0.01 M sodium phosphate buffer solution (pH 7.4) were prepared. Both suspensions were mixed together. The concentration of nanotraps in the final suspension was 0.50 mg/mL while the concentration of VMPs was 0.15 mg/mL. The mixture was incubated at 25 °C for 30 min. After 5, 10, 20, and 30 min the mixture was analyzed by DLS and NTA to monitor the aggregation process.

### 2.9. Theoretical Calculations

The specific surface area of nano- and microparticles as well as the number of protein molecules per nanoparticle was calculated as described in the Electronic Supporting Information to the article published by Kritchenkov et al. [29]. A brief description of theoretical calculations used in this work is given in Supplementary Materials.

## 3. Results and Discussion

### 3.1. Production of the LEL CD81-SA Fusion Protein

**Figure 2:**
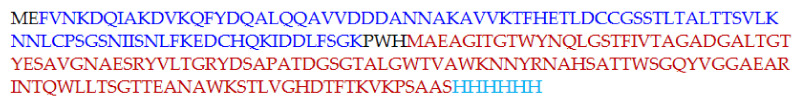
The amino acid sequence of the recombinant chimeric protein LEL CD81-SA. The blue color indicates the amino acid sequence of LEL (116–202 a.a.) of the CD81 protein, and the red color indicates the amino acid sequence of the SA protein.

As it was mentioned earlier, CD81 plays an important role in the attachment of the hepatitis C virus to the host cell through the interaction of protein large extracellular loop with the viral protein E2. In order to prepare trapping system capable of HCV binding, a recombinant LEL CD81 fusion protein with streptavidin was created as a receptor for immobilization onto the PLA microparticles. For that, initially, we designed a genetic construction expressing the nucleotide sequence of streptavidin with LEL CD81. The strain of *E. coli* DH5(α) was used to develop the initial vector of pET SA 2-7. To insert the nucleotide sequences, the “sticky ends” obtained by processing the vector with the NdeI restriction endonuclease were used. The source vector was then used to obtain a genetic construct encoding streptavidin with LEL CD81. This genetic construct encoding streptavidin with CD81 was transformed into the *E. coli* strain BL21 (DE3), which was cultivated further as described in the Experimental part. The amino acid sequence of the resulting protein is shown in Figure 2.

**Figure 3:**
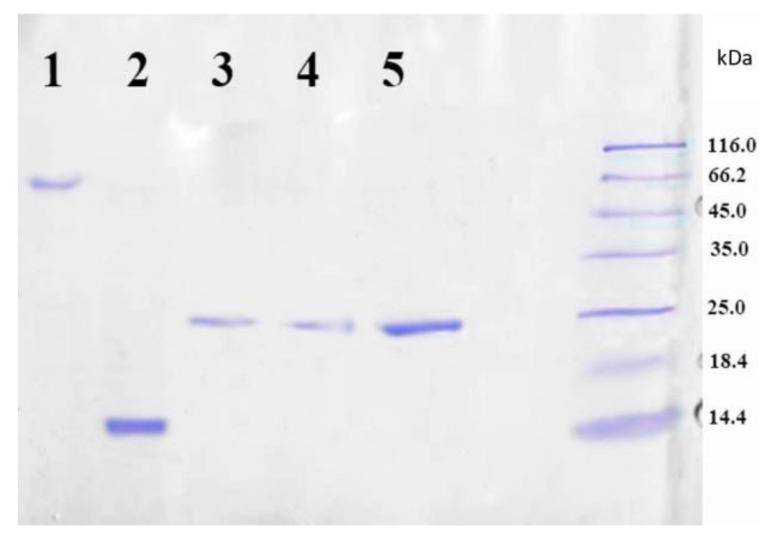
SDS PAGE analysis of purified LEL CD81-SA: lane 1—SA solution boiled with addition of SDS, lane 2—SA solution boiled with addition of SDS and 2-mercaptoethanol, lane 3—LEL CD81-SA solution boiled with addition of SDS, lane 4—LEL CD81-SA solution boiled with addition of SDS and 2-mercaptoethanol, lane 5—LEL CD81-SA filtered through the membrane with MWCO of 50,000. Coomassie R-250 Staining.

The target LEL CD81-SA protein was isolated from the inclusion bodies and the soluble cell fraction. The presence of protein in the fractions was testified by SDS PAGE method. Considering that native streptavidin is a tetrameric protein, it was assumed that the fusion protein LEL CD81-SA could have also a tetrameric structure due to the formation of the bonds between the SA fragments. If this was a case, the molecular weight of the fusion protein would have to be approximately 100 kDa. To prove or disprove this assumption, the SDS PAGE was carried out both without pre-boiling and addition of β-mercaptoethanol (non-denaturing technique), and with pre-boiling at 100 °C and the addition of β-mercaptoethanol (denaturing technique). Figure 3 shows the results of gel electrophoresis analysis of the LEL CD81-SA protein isolated from inclusion bodies. One can see that both samples, namely the sample loaded into gel without denaturation (lane 3 and 5) as well as denaturized one (lane 4), gave the same results on the molecular weight. In both cases, the molecular weight was determined to be around 25 kDa. Thus, the obtained fusion protein LEL CD81-SA is monomeric, but not tetrameric like the native streptavidin (SA).

### 3.2. Production of the E2-GFP Fusion Protein

Currently, the recombinant E2 core expressed in human cell culture is widely used for model studies [2,30]. It is established that such protein binds to human CD81 protein [21,22,31]. In our case, we developed an approach to obtain the minimal E2 core fragment (412–645) in the non-glycosylated form and used it for the preparation of HC VMPs and the study of their binding with developed PLA-based LEL CD81-SA trapping systems.

E2 core is slightly shorter than the full-length E2 protein, and according to the literature [21], this is the minimum site required for interaction with CD81. In our first attempt, we started with the synthesis of a fragment of 412–645 a.a. of E2 core in *E. coli* (Supplementary Materials). The resulting protein was accumulated in the inclusion bodies and could be dissolved only in a strong detergent (8 M urea). The different attempts for renaturation were not successful since the target protein remained aggregated, and after 1–4 h it precipitated. A similar result was previously published by Yurkova et al. [32], who described the recombinant synthesis of a slightly different fragment (385–663 amino acid residues) of E2 in *E. coli*. The authors also noted that the target protein aggregated at all the conditions they tested even after renaturation.

**Figure 4:**
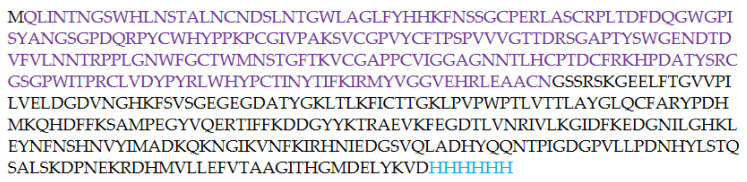
Amino acid sequence of the E2-GFP fusion protein. E2 core (412–645) amino acid sequence is colored in violet.

In order to develop a soluble form of E2 protein, we decided to synthesize a fusion form of E2 core and GFP (Figure 4). A genetic expression construct representing a plasmid based on pTrc99A, which includes the E2 core and the superfolder GFP genes in one reading frame, was created. In many cases, the conjugation with GFP allows the preparation of a soluble target product in *E. coli*, while the same product without superfolder GFP aggregates and is often unsuitable for further experiments [33,34]. On the one hand, superfolder GFP increases the solubility of the associated protein. On the other hand, the “green” part of the chimeric protein increases the convenience of manipulation with the protein due to its ability to visualization both by fluorescent confocal microscopy and just by eyes. The nucleotide sequence of the plasmid was composed in such a way that the C-terminus of the E2-GFP chimeric protein synthesized in *E. coli* had a 6-His tag for further affinity purification (Figure 4). The resulting plasmid was inserted into BL (DE3) *E. coli* expression strain. The synthesis was carried out as described in Experimental part.

**Figure 5:**
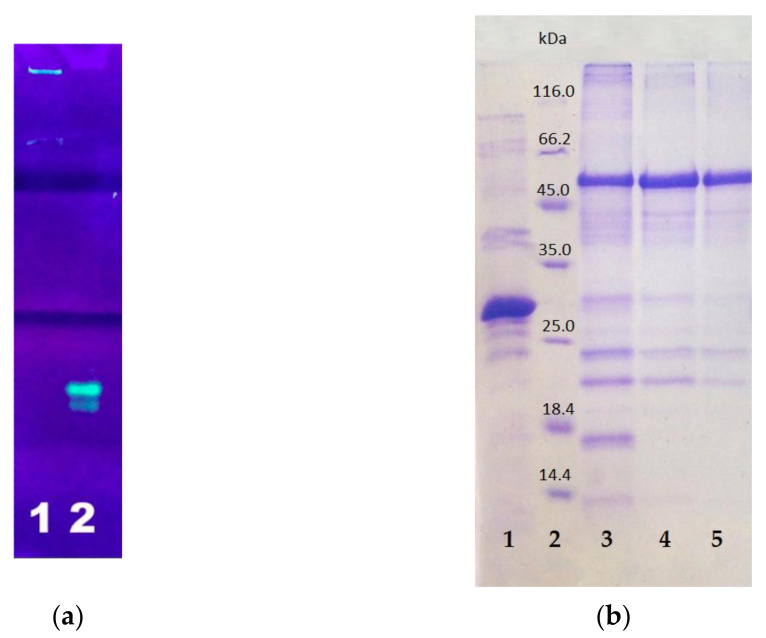
PAGE analysis of E2-GFP isolated from inclusion bodies: (**a**) non-denaturing PAGE with fluorescence detection: lane 1—E2-GFP, lane 2—GFP; (**b**) SDS PAGE with Coomassie staining: lane 1—GFP; lane 2—protein markers; lanes 3–5—E2-GFP from different fractions.

After the synthesis was completed, the cells were destroyed by sonication and centrifuged to separate the soluble and insoluble cell fractions. The insoluble cell fraction, usually containing inclusion bodies, was dissolved in 8 M urea. Both fractions were purified by affinity chromatography on Ni-agarose column. In both cell fractions, the resulting E2-GFP had green fluorescence. The target E2-GFP was detected both in the soluble cell fraction (<100 µg/L of culture medium) and in the inclusion bodies (3–10 mg/L of culture medium). Since the amount of the target protein was much higher in the inclusion bodies, the isolation from this source was preferable. SDS PAGE analysis in 5–12% polyacrylamide gel testified that the synthesized target E2-GFP fusion protein, isolated from the inclusion bodies, remained full-sized (54 kDa) (Figure 5).

The proposed method of renaturation of the E2-GFP of using step-wise dialysis (see Section 2.3) makes it possible to obtain a product soluble in aqueous media and that is able to bind to the CD81 receptor. Besides the improvement of the E2 solubility, the fluorescence of GFP, conjugated with E2, is very useful to study the binding of LEL CD81-SA with E2.

### 3.3. Study of the Interaction between CD81-SA and E2-GFP Proteins

**Figure 6:**
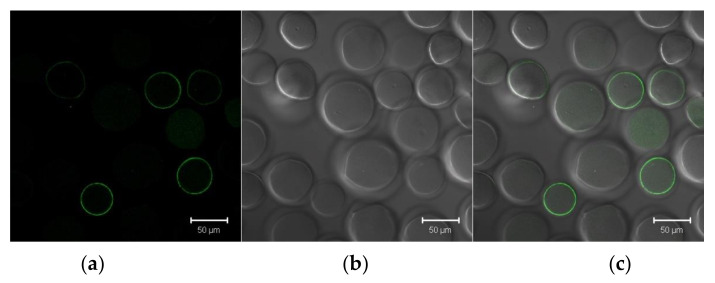

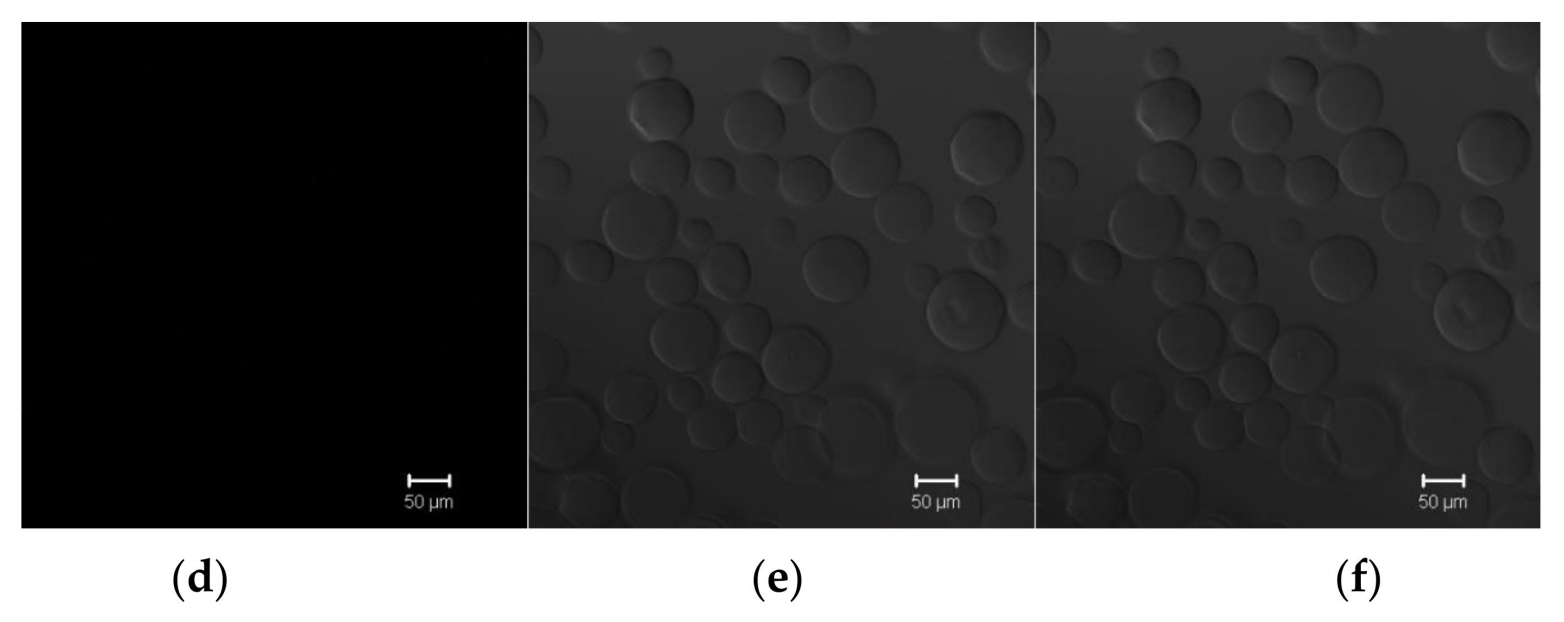
Confocal microscopy images of the Sepharose beads modified with LEL CD81-SA and contained affinity bound (**a**–**c**) or eluted (**d**–**f**) E2-GFP. Images (**a**,**d**) registered in the fluorescent mode (excitation at 488 nm); (**b**,**e**)—differential interference contrast (DIC); and (**c**,**f**) represent the combined images of (**b**,**c**), and (**d**,**e**), respectively.

The ability to affinity binding of the synthesized LEL CD81-SA to E2 is a principal issue to develop effective HCV trapping system. In order to study the binding of LEL CD81-SA with E2-GFP, the first one was covalently immobilized onto the Sepharose beads using the intermediate activation of Sepharose with CNBr. The solution of E2-GFP in PBS was loaded onto the column which was washed to remove free and non-specially bound protein. After that, the aliquot of the Sepharose beads was taken and analyzed by confocal microscopy (Figure 6a–c).

It was found that about 90% of Sepharose beads had a weak green fluorescence and about 50% of beads were covered with the protein on their surface. After the E2-GFP elution with 0.3 M Tris-glycine buffer, pH 9.5, the Sepharose beads did not demonstrate any green fluorescence (Figure 6d–f). Thus, both synthesized proteins were capable of binding to each other. Moreover, the E2-GFP protein, stored at 6 °C with the addition of sodium azide during a month, did not change its electrophoretic mobility and retained its affinity to LEL CD81-SA.

### 3.4. Preparation of HC Micro- and Nanotraps and HC VMPs

Two kinds of polymer particles based on PLA and differing in size were applied for the preparation of HCV trapping systems. PLA was selected since it is a biodegradable, biocompatible, non-toxic and FDA approved polymer for application in vivo [35]. To prepare micro- and nanotraps for HCV, both polymer micro- and nanoparticles were functionalized with LEL CD81-SA by the covalent immobilization. Microparticles with average diameter of 800 or 1640 nm were obtained by single emulsion technique from pre-synthesized PLA while nanoparticles with average diameter of 90 nm were prepared by nanoprecipitation technique from pre-synthesized PEG-*b*-PLA. Due to their large size, microtraps are very convenient objects for capturing HCV in vitro since they can be easily centrifuged and separated. At the same time, they are poorly suitable for in vivo application. The large size of microtraps makes them recognizable for macrophages, which can be a reason for their faster elimination from the blood stream compared to nanotraps [36]. In turn, PEGylated PLA-based nanotraps in their neat state are less visible to macrophages, but an increase in their size after binding to HCV will contribute to better recognition and elimination due to phagocytosis.

**Figure 7:**
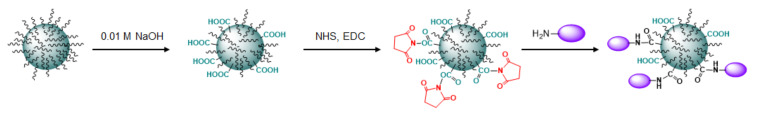
Scheme for modification of PEG-*b*-PLA nanoparticles with protein (LEL CD81-SA for preparation of nanotraps or E2-GFP for VMPs’ obtaining). The same approach was applied for the modification of PLA microparticles with LEL CD81-SA to prepare microtraps.

In both cases, the immobilization of LEL CD81-SA was fulfilled via the reaction of amino groups of protein with activated carboxylic groups generated at the surface of polymer particles as a result of treatment with sodium hydroxide solution (Figure 7). The characteristics of initial micro- and nanoparticles as well as their forms functionalized with corresponding proteins are summarized in Table 1. In addition, PLA-based microparticles were functionalized with SBTI-Cy3 as a model protein to obtain a negative control for evaluating binding selectivity. Since nanoparticles are much smaller than microparticles and have higher total specific surface area (approx. 53 m^2^·g^−1^ for nanoparticles and 6 m^2^·g^−1^ for microparticles), they were able to immobilize the higher amount of protein. In particular, 5–7 μg of protein per mg of microparticles was the highest amount of protein that we could immobilize. In turn, it was easy to immobilize up to 70 μg of protein per mg of nanoparticles.

**Figure 8:**
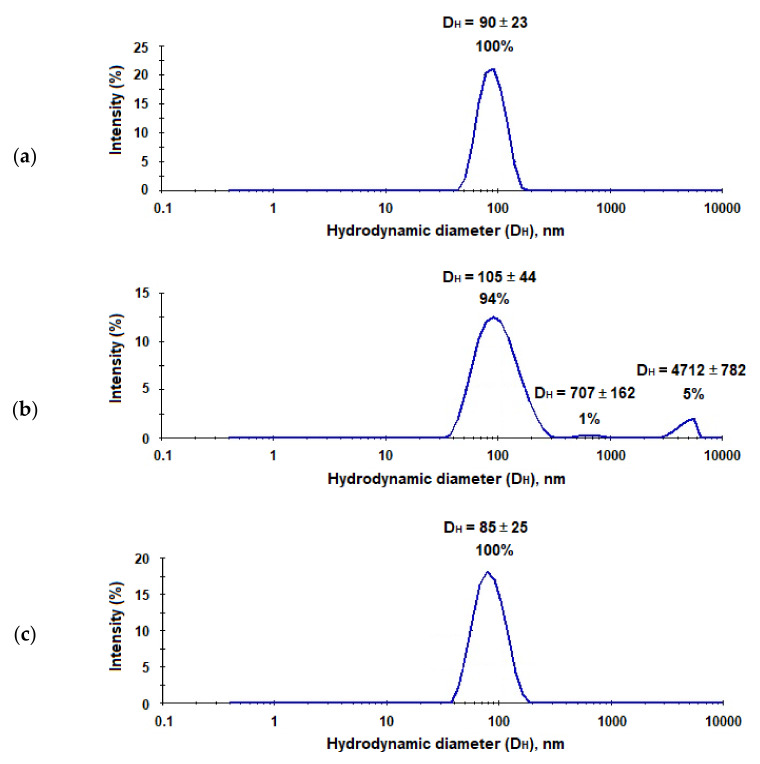
Results of DLS analysis of the neat PEG-*b*-PLA nanoparticles (**a**), PEG-*b*-PLA modified with the LEL CD81-SA-Cy3 conjugate (nanotraps) (**b**), and PEG-*b*-PLA modified with E2-GFP (HC VMPs) (**c**).

In order to prepare hepatitis C virus-mimicking particles (HC VMPs), we used nanoparticles with a size close to HCV, namely, to 90 nm [23], and modified them with E2-GFP using the same way as for the preparation of nanotraps. The characteristics of system obtained are collected in Table 1. It was found that the modification of micro- and nanoparticles with proteins was only slightly affected by the hydrodynamic diameter of polymer particles, while PDI change was more pronounced. However, if PDI for microparticles has become slightly lower, for nanoparticles this parameter has increased significantly. The most considerable increase in PDI was observed for nanoparticles modified with LEL CD81-SA-Cy3 (Table 1, Figure 8). Moreover, for this system, the appearance of small amount (6%) of aggregates was also detected by DLS. In all cases, after modification, the surface kept negative charge, which provides high stability towards the aggregation in time. The storage of both nanotraps and VMPs for a month did not follow by any change of their characteristics.

### 3.5. Study of the Interaction between HC Trapping Systems and E2-GFP/HC VMPs

**Figure 9:**
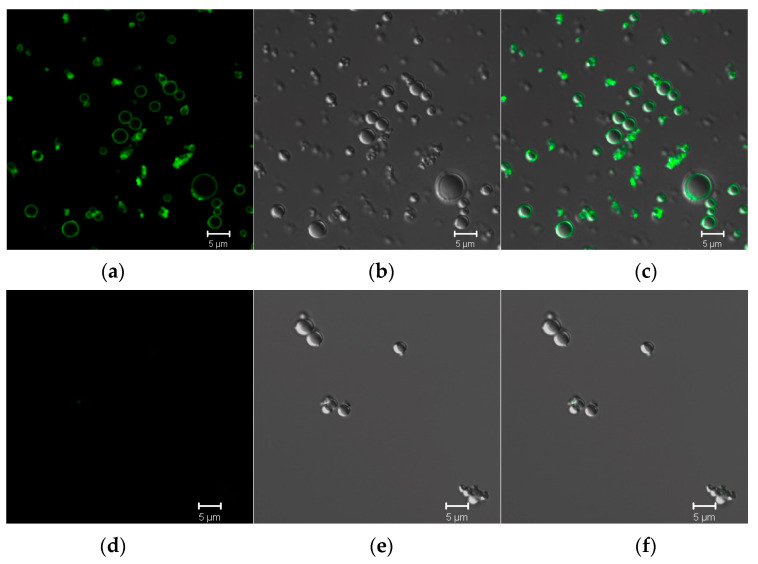
Confocal microscopy images of the microtraps incubated with E2-GFP (**a**–**c**) and GFP as negative control (**d**–**f**). Images (**a**,**d**) registered in the fluorescent mode (excitation at 488 nm); (**b**,**e**)—differential interference contrast (DIC); and (**c**,**f**) represent the combined images of (**b**,**c**), and (**d**,**e**), respectively.

The ability of the developed HC trapping systems to bind selectively E2 protein was evaluated with the use of E2-GFP as a target protein and GFP as negative control. Both proteins were incubated with developed HC microtraps, which were then washed twice and analyzed by confocal microscopy. It was established that the surface of microtraps incubated with E2-GFP demonstrated green fluorescence after excitation under a light with a wavelength of 488 nm (Figure 9a–c). At the same time, the surface of microtraps incubated with control GFP under the same conditions had no fluorescence (Figure 9d–f). Thus, the developed LEL CD81-conaining microtraps were capable of efficient and selective binding of E2 protein, which is the main antigen protein of hepatitis C.

**Figure 10:**
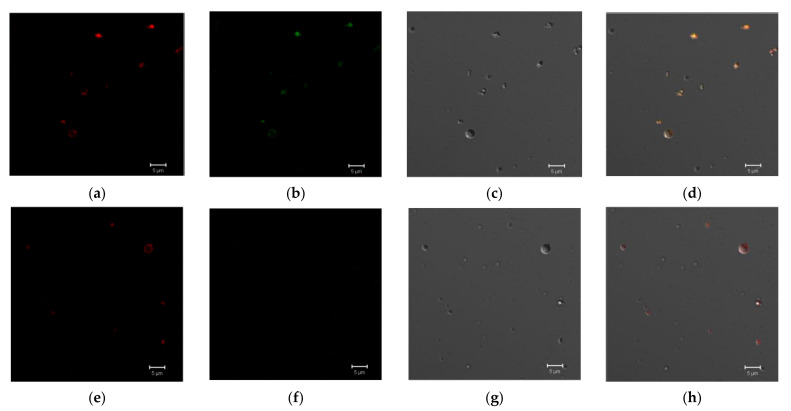
Confocal microscopy images of the microtraps modified with Cy3 and incubated with E2-GFP (**a**–**d**) and microparticles modified with SITR-Cy3 and incubated with E2-GFP (**e**–**h**). Images (**a**,**e**) and (**b**,**f**) registered in the fluorescent mode for Cy3 and GFP, respectively; (**c**,**g**)—differential interference contrast (DIC); and (**d**,**h**) represent the combined images of (**a**–**c**) and (**e**–**g**), respectively.

The selectivity of E2 binding to LEL CD81-SA was also evaluated. In this case, to bind E2-GFP, we used PLA microparticles modified with SBTI labeled with Cy3 as a negative control and microtraps containing LEL CD81-SA also labeled with the same dye. The polymer microparticles were detected using red fluorescence of Cy3 (Figure 10a,e) while their binding to E2-GFP was registered via GFP green fluorescence (Figure 10b,f). Thus, as in previous experiments, the microtraps containing CD81-SA successfully bound E2-GFP. At same time, the microparticles bearing control protein did not show any green fluorescence that, in turn, means an absence of E2-GFP binding. Thus, it can be deduced that the synthesized LEL CD81-SA was capable of selective binding E2-GFP and vice versa.

**Figure 11:**
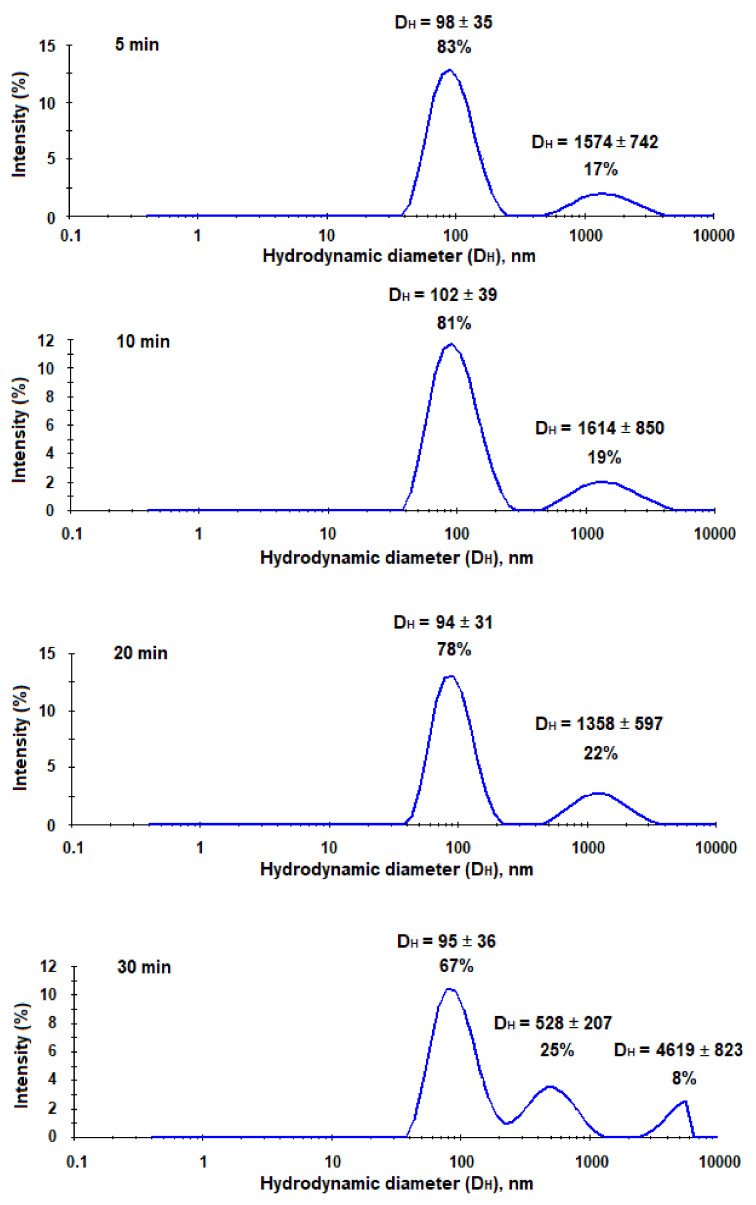
Monitoring of interaction between nanotraps and HC VMPs during time (DLS analysis).

As it was mentioned earlier, the trapping systems for the application in vivo should be quite small, stable, and have a reduced uptake by macrophages. It is known, that PEG-*b*-PLA nanoparticles have a good stability due to additional surface hydrophilization and lower uptake by microphages than PLA nanoparticles [37]. Moreover, PEGylation is also well-known approach to reduce non-specific interaction with plasma proteins [38]. However, after capturing HCV, nanotraps should increase in their size and become recognizable for macrophages in vivo. In order, to evaluate the increase of nanotraps in size after their interaction with hepatitis C virus-mimicking particles, we incubated the suspension of HC VMPs in 0.01 M sodium phosphate buffer solution (pH 7.4) with the suspension of nanotraps in the same buffer. The characteristics of the system were monitored by DLS for 30 min. As seen from Figure 11, the aggregation started immediately and progressed during experiment time. In particular, after 5 min of co-incubation one can see the appearance of additional mode corresponding to large aggregates. After 10 and 20 min of co-incubation the percentage of aggregates increased among with the decrease of mode corresponding to nanoparticles. After 30 min the appearance of the third mode was detected. In total, the percentage of aggregates was equal to 33%.

**Figure 12:**
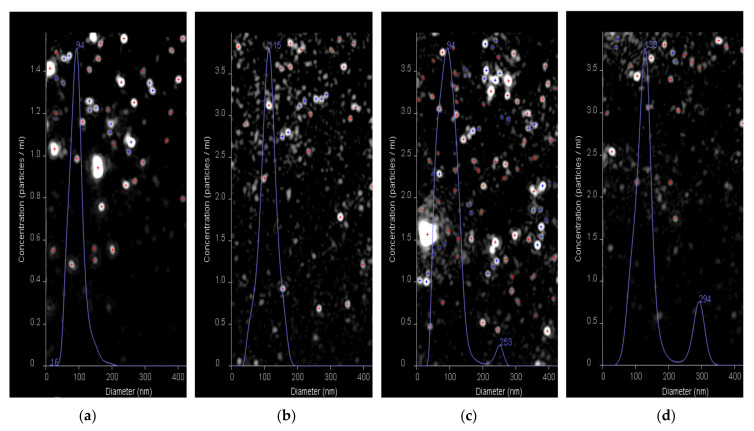
Results of nanoparticle tracking analysis (NTA) of HC VMPs (**a**), nanotraps (**b**) and interaction between nanotraps and HC VMPs (**c**,**d**) for 5 and 20 min, respectively.

Additionally, the nanotraps and HC VMPs obtained as well as their mixture were evaluated using nanoparticle tracking analysis (NTA) (Figure 12). The average hydrodynamic diameter for the HC VMPs and nanotraps were detected to be 94 and 115 nm, respectively (Figure 12a,b). The *D_H_* values measured by NTA were in agreement with those determined by DLS, namely 90 and 105 nm, respectively. The coincubation HC VMPs with nanotraps revealed the appearance of the second mode corresponding to the particles with the average diameter in the range of 250–300 nm (Figure 12c,d). Since these large particles were not present in the initial samples of HC VMPs and nanotraps it could be assumed that they are aggregates. The particles of larger size were not detected in the samples. Nevertheless, since the NTA method is suitable for the analysis of objects with size up to 500 nm (due to limited capillary availability for larger nanoparticles), it is definitely impossible to exclude the absence of the larger aggregates in the mixture. However, such aggregates could be detected by DLS (Figure 11). Thus, the evidence for formation of aggregates between nanotraps and HC VMPs was provided by both DLS and NTA methods.

The calculation of the number of protein molecules’ bound per nanoparticle (see Section 2.9 and Supplementary Materials) was carried out to evaluate the average number of HC VMPs which can be captured by each nanotrap. The approximate number of protein molecules immobilized per nanoparticle was equal to 1.7 in the case of nanotraps (LEL CD81-SA immobilization) and 0.8 in the case of HC VMPs (E2-GFP immobilization). Thus, taking into account that HC VMPs binding to nanotraps is based on the interaction between proteins immobilized on the surfaces of these particles, one can suppose that in an averaged case, each nanotrap can capture two HC VMPs. However, since the protein immobilization on the surface of nanoparticles is a random process, the composition of aggregates can vary.

The effect of nanoparticles’ size on their cellular uptake, biodistribution and pharmacokinetics were studied in a number of works [36,39,40,41]. For instance, recently Churilov et al. studied the phagocytosis of camel IgG or BSA-modified delivery systems of 4-thioureidoiminomethylpyridinium perchlorate based on PLA micro- and nanoparticles using macrophages derived from the peritoneal cavity of mice [42]. They found that uptake of microparticles by macrophages was more extensive than that for nanoparticles. De Jong et al. evaluated the intravenous administration of gold nanoparticles with a diameter of 10, 50, 100, and 250 nm in rats and found that smaller nanoparticles demonstrated better biodistribution than large due to their faster elimination from the blood stream [43]. A similar tendency was also observed by Feng et al. for PEGylated polymer particles. In particular, the authors studied the nanoparticles of 80, 170, and 240 nm in diameter and found the uptake of PEGylated polymer nanoparticles was size dependent. The highest uptake was observed for 240 nm nanoparticles while the longest circulation time was detected for 80 nm ones. The faster blood clearance for the larger particles was also testified by Koide et al. [44]. The authors compared the administration of PEGylated liposomes (119, 261 and 795 nm) with polymeric micelles (ca. 10, 32 and 50 nm) in mice. The accelerated clearance was detected for PEGylated liposomes in comparison to smaller polymer micelles. Thus, the published findings allow the presumption that the increasing of nanotraps in size after capturing the virus will favor better elimination of such aggregates in vivo in comparison with native virus particles and neat nanotraps.

## 4. Conclusions

The fusion protein consisted of LEL CD81 as capturing moiety for HCV and streptavidin as spacing part was designed and synthesized. In contrast to native streptavidin, which is a tetrameric protein, the obtained LEL CD81-SA conjugate contained monomeric SA. The protein obtained was successfully immobilized onto the surface of PLA microparticles and PEG-*b*-PLA nanoparticles. The obtained systems were stable and easily bound to E2 core protein of the hepatitis C virus. Thus, the developed systems can potentially serve as micro- and nanotraps for the hepatitis C virus both in vitro for diagnostic purposes and in vivo to reduce the acute viremia, respectively. The offered approach for the preparation of micro- and nanotraps can be a basis for the development of the nanotherapeutics for both hepatitis C and other viral infections.

## Figures and Tables

**Table 1:** Characteristics of micro- and nanoparticles, hepatitis C trapping systems and virus-mimicking nanoparticles.

System	Polymer Particles	Protein for Immobilization	Characteristics of Neat Polymer Particles (in PBS)			Characteristics of Modified Polymer Particles (in PBS)			
			*D_H_*, nm	PDI	ζ-Potential	*D_H_*, nm	PDI	ζ-Potential	Amount of Bound Protein, µg/mg of Particles
Microtraps	PLA	LEL CD81-SA-Cy3	800	0.39	−34	850	0.26	−68	3
			1640	0.37	−38	1860	0.28	−48	2
Negative control	PLA	SBTI-Cy3	800	0.39	−34	860	0.23	−75	2.5
Nanotraps	PEG-*b*-PLA	LEL CD81-SA-Cy3	90	0.05	−24	105	0.29	−43	15
HC VMPs	PEG-*b*-PLA	E2-GFP	90	0.05	−24	85	0.19	−35	15

## Supplementary Materials

The following are available online at https://www.mdpi.com/article/10.3390/pharmaceutics13050672/s1, Figure S1: Scheme of creation of pET SA2-7 (a) and pET SA 2-7–CD81 (b) genetic constructs for production of CD81-SA fusion protein; Methods for Synthesis E2 in E. coli (first attempts); Calculation of the Specific Surface Area and Number of Bound Protein Molecules per Nanoparticle.
