# Supplementary material for: Polymer Particles Bearing Recombinant LEL CD81 as Trapping Systems for Hepatitis C Virus
Source: Pharmaceutics. 2021 May 7;13(5):672. doi: 10.3390/pharmaceutics13050672 (PMC8151308; doi:10.3390/pharmaceutics13050672)
Supplement: Supplementary file 1 [file pharmaceutics-13-00672-s001.zip › pharmaceutics-1203573-supplementary.pdf]

pharmaceutics

Supplementary Materials: Polymer Particles Bearing Recombinant LEL CD81 as Trapping Systems for Hepatitis C Virus

Dmitry Polyakov, Ekaterina Sinitsyna, Natalia Grudinina, Mariia Antipchik, Rodion Sakhabeev, Viktor Korzhikov-Vlakh, Mikhail Shavlovsky, Evgenia Korzhikova-Vlakh and Tatiana Tennikova

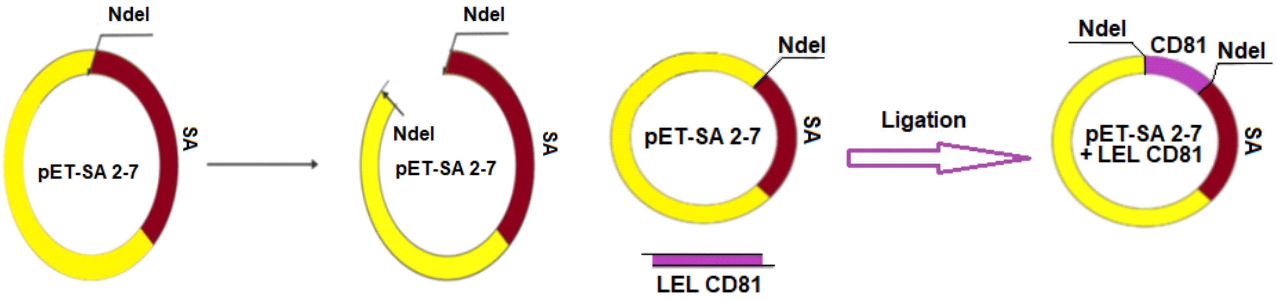

(a) **(b)**

Figure S1. Scheme of creation of pET SA2-7 (**a**) and pET SA 2-7–CD81 (**b**) genetic constructs for production of CD81-SA fusion protein.

Methods for Synthesis E2 in E. coli (first attempts)

Initially, the full-length E2 (amino acid sequence 384–746) was synthesized in *E. coli.* For this, the cDNA encoding the E2 sequence (genotype 1a, isolate 1, HCV) was inserted into plasmid pET22b (+) (Novagen) at the NdeI and XhoI restriction sites. Oligonucleotides 5′-ctgcatatggCAGCTGATCAACACC-3′ и 5′-ctggtcgacGTTGCAAGCAGCTTC-3′ were utilized for preparation of PCR-product of 717 b.p. The obtained plasmid was sequenced and inserted into *E. coli* cells of the expression strain BL (DE3). The clone grown on selective agar LB medium was placed in 10 mL of LB medium containing 100 μg/mL of ampicillin and then grown overnight at 37 °C under aeration with constant stirring. Then, this inoculum was placed into 16 flasks of 250 mL of fresh LB medium (with the addition of ampicillin), and the cells were grown to optical density equal to 0.8 (λ = 600 nm). After that, each 4 flasks were incubated at 20, 26, 30 and 37 °C, respectively. Induction of protein synthesis was performed for 16 h with the addition of 1.0, 0.5, 0.25 or 0 mmol of isopropyl-β-D-1-thiogalactopyranoside (IPTG). The cells were centrifuged, washed with saline solution, destroyed by ultrasonication, and then centrifuged to separate the soluble and insoluble cell fractions. The insoluble cell fraction, usually containing inclusion bodies, was dissolved in 8M urea.

To synthesize E2 in *E. coli* with the accumulation of the target protein in the periplasm, the gene encoding E2 sequence 412-645 was inserted into plasmid pET22b (+) at the NcoI and XhoI restriction sites. The general procedure was similar to previous but the direct primer was 5′-ctggaattcgCAGCTGATCAACACC-3′. The design of the plasmid was made in such a way that the encoding portion of E2 was followed with a sequence encoding of 6-His necessary for affinity purification of the protein on nickel-agarose gel. Recombinant E2 was prepared in *E. coli* of strain BL21 (DE3) transformed with the described expression genetic construct. *E. coli* grew overnight in 10 mL of LB medium containing 1 mg of ampicillin and then were introduced into 500 mL of fresh medium. The

cultivation was carried out under aeration conditions at 37 °C until an optical density was 0.8 (λ = 600 nm). The synthesis of the desired protein was performed in presence of 0.5 mM IPTG overnight at 26 °C under aeration conditions. A similar experiment was also carried out with an IPTG concentration of 1 mM. After separation of the bacterial cells by centrifugation at 10,000 g, the periplasmic fraction was obtained by the "osmotic shock" method.

Such a strategy is sometimes used to avoid the aggregation of the synthesized product in the insoluble inclusion bodies and, as result, to obtain a soluble target protein. In this case, specific bacterial leader peptides are used to guide the synthesized protein into the periplasm of the bacterium. After passing through the cell membrane, the leader peptide is usually cut off by a special bacterial protease. In addition, intramolecular S-S bonds are usually closed in the periplasmic space. Considering that the E2 protein of interest should have 3 disulfide bonds, and also considering the failures with the renaturation of the protein from the inclusion bodies, we created an expression genetic construct for the synthesis of E2 in the periplasm of *E. coli.* For this purpose, a pelB leader peptide was used. The presence of synthesized E2 was checked by affine isolation of the contents of the periplasm on a metal-chelated Ni-agarose sorbent. The fractions were analyzed by electrophoretic separation in 5–12% polyacrylamide gel under denaturing conditions with following Coomassie staining. However, the target protein was not detected.

Calculation of the Specific Surface Area and Number of Bound Protein Molecules per Nanoparticle

Briefly, the specific surface area was calculated using the following equation:

$$S = 6 / (\rho \cdot D) \qquad (1)$$

where S is a specific surface area ($m^2 \cdot kg^{-1}$), ρ is a density of polymer ($kg \cdot m^{-3}$) and D is a diameter of polymer particle (m). For calculations ρ of poly(D,L-lactic acid) was taken as 1.25 $kg \cdot m^{-3}$ [1] and D was taken as 90 nm and 800 nm for nano- and microparticles, respectively. For PEG-*b*-PLA the ρ was taken also as 1.25 $kg \cdot m^{-3}$ since the PLA block is much longer than PEG, and the density of used PEG-5000 is close to the density PLA (1.21 $kg \cdot m^{-3}$).

The number of nanoparticles in a sample was calculated as follows:

$$N_n = m_s / m_p \qquad (2)$$

where N is number of particles, m_s is a mass of nanoparticles in the sample (g), m_p is a mass of single nanoparticle (g) calculated using the equation:

$$m_p = V_{sphere} \cdot \rho = 4/3 \cdot \pi \cdot r^3 \cdot \rho \qquad (3)$$

where V_{sphere} is a volume of sphere (m^3), ρ is a density of polymer ($kg \cdot m^{-3}$) and r is a radius of polymer particle (m).

The number of protein molecules bound to the nanoparticles were calculated as follows:

$$N_{p/n} = N_p / N_n \qquad (4)$$

where $N_{p/n}$ is a number of protein molecules bound per single nanoparticle, N_n is a number of nanoparticles in the sample; N_p is a number of protein molecules bound to the nanoparticles calculated using the equation:

$$N_p = (m \cdot N_A) / M \qquad (5)$$

where m (g) and M ($g \cdot mol^{-1}$) are the mass and molar weight of the bound protein, respectively, N_A is the Avogadro's number.

References

1. Li, X.; Murthy, N.S.; Latour, R.A. Structure of hydrated poly(D,L-lactic acid) studied with X-ray diffraction and molecular simulation methods. *Macromolecules* **2012**, *45*, 4896–4906.
